# Gender bias in clinical case reports: A cross-sectional study of the “big five” medical journals

**DOI:** 10.1371/journal.pone.0177386

**Published:** 2017-05-11

**Authors:** Pascale Allotey, Caitlin Allotey-Reidpath, Daniel D. Reidpath

**Affiliations:** 1 Jeffrey Cheah School of Medicine and Health Sciences, Monash University Malaysia, Bandar |Sunway, Malaysia; 2 College of Medicine & Veterinary Medicine, University of Edinburgh, Edinburgh, United Kingdom; Royal College of Surgeons in Ireland, IRELAND

## Abstract

**Background:**

Gender bias in medical journals can affect the science and the benefit to patients. It has never been investigated in clinical case reports. The oversight is important because of the role clinical case reports play in hypothesis generation and medical education. We investigated contemporary gender bias in case reports for the highest ranked journals in general and internal medicine.

**Methods:**

PubMed case reports data from 2011 to 2016 were extracted for the *Annals of Internal Medicine*, *British Medical Journal*, the *Journal of the American Medical Association*, *The Lancet*, and *New England Journal of Medicine*. The gender of the patients were identified and a text analysis of the Medical Subject Headings conducted.

**Results:**

A total of 2,742 case reports were downloaded and 2,582 (95.6%) reports contributed to the final analysis. A pooled analysis showed a statistically significant gender bias against female case reports (0.45; 95%CI: 0.43–0.47). The *Annals of Internal Medicine* was the only journal with a point estimate (non significant) in the direction of a bias against male patients. The text analysis identified no substantive difference in the focus of the case reports and no obvious explanation for the bias.

**Conclusion:**

Gender bias, previously identified in clinical research and in clinical authorship, extends into the patients presented in clinical case reports. Whether it is driven by authors or editors is not clear, but it likely contributes to and supports an overall male bias of clinical medicine.

## Introduction

The late 1980s saw an acceleration in efforts to highlight and address the gender bias in science and medical research.[1,2] There are issues of justice associated with such a bias, and there are significant empirical concerns about the generalizability of gender biased findings and their applicability to clinical practice.[3] The efforts to address the imbalance has resulted in the establishment of several peak bodies to address gender in science, targeted funding streams to enhance both opportunities for women scientists and extend women's participation in research, and policies that require researchers to address specifically the gender implications of their design, recruitment strategies and findings. A recent “call for action” is seeking to include gender in research impact assessment.[4] Similarly, key biomedical journals increasingly require gender reporting.[2,5]

A recent review of progress on gender equality was undertaken across a range of indicators. including on authorship of research papers. The prevalence of female first authors in major medical journals increased from 27%-37% between 1994–2014, but this result was not consistent across journals.[6] We sought to investigate the gender gap further with a focus on the gender bias in clinical case reports.

“The [clinical] case report is the archetypical medical article”, at least that was a view expressed in JAMA in 1968.[7] Today, the case report is less common, having been overtaken by research papers of experimental and quasi-experimental studies. Nonetheless the clinical case report maintains a key role in the medical literature as a vehicle for reporting unusual disease presentations or outbreaks,[8,9] as a hypothesis generator,[10,11] and most typically as a pedagogic tool.[12–14] In their “Instructions to Authors”, for example, the *British Medical Journal*, and the *Lancet* highlight the place of the case report in this latter role. The *British Medical Journal* mentions the requirement for “real cases … suitable for presentation in specifically educational formats”, and the *Lancet* observes that “novelty is not essential, but at least one broadly useful learning point is.”

A case report could be about any patient or any clinical condition, and it is unreasonable to expect that a single case report is gender balanced. In their specificity they are necessarily biased. As a corpus of case reports within a general and internal medicine journal, however, one would hope to see a representative sample of the patient population, gender balanced.

## Materials and methods

### Search strategy

We selected the top five ranked journals, by impact factor (IF), in general and internal medicine for the review (the “big five”): *Annals of Internal Medicine* (IF: 16.6); *British Medical Journal* (IF: 19.7)*; Journal of the American Medical Association* (IF: 37.7)*; The Lancet* (IF: 44.0)*;* and *New England Journal of Medicine* (IF: 59.6).[15] We did not go beyond those five journals, because of their place and impact in medicine; and the sixth ranked journal, *PLoS Medicine*, does not publish case reports. We focused the search on case reports since 2011 to capture contemporary rather than historical editorial/authorial behavior.

PubMed was the only on-line data base searched because the journals’ articles are completely indexed in the single database. The search terms combined each journal's PubMed abbreviation for its name (e.g., “JAMA” for the *Journal of the American Medical Association* and “Ann Intern Med” for the *Annals of Internal Medicine)* with the case reports as the type of article (“Case Reports[ptyp]”) limited to the five years from 01 January 2011 to 02 September 2016 ("2011/01/01"[PDAT]: "2016/09/02"[PDAT]). The complete *Lancet* search, for example, was:

"Lancet"[Journal] AND (Case Reports[ptyp] AND "2011/01/01"[PDAT]: "2016/09/02"[PDAT])

The retrieved records from the searches formed the data for the subsequent analyses. The Medical Subject Headings (MeSH) were used to identify the sex of the individual(s) described in the case report.

### Analysis

We defined a bias in case reports in favour of one or other gender by a proportion of female case reports significantly different from 0.5. The proportion and 95% confidence intervals of case reports relating to female patients were estimated for each journal. A mixed effects logistic model was estimated with random intercepts and fixed effect for the journals. Finally the pooled data was used to estimate the proportion and 95% confidence intervals of case reports relating to female patients.

We took into account that a gender bias in case reports might be explicable by the type of clinical condition being reported. A text analysis of the MeSH of the case reports was therefore conducted. Specifically, the MeSH within each case report were combined and any repeat words were removed. By gender, the MeSH text for all case reports were combined and a count made of the occurrence of each word. For example, if “neoplasm” occurred twice in a single case report’s MeSH only one occurrence was counted. The total count for “neoplasm” reflected the number of case reports in which the word occurred at least once. Two of the authors (PA and DDR) independently categorised the most frequently occurring words into one of four categories: Body/Organ; Disease; Symptom; Investigation/Treatment. Disagreements were resolved by discussion and the results tabulated.

Bibliographic details of individual case report were downloaded from PubMed using the RISmed package in the R statistical environment.[16,17] With the exception of the final classification of MeSH words, all analyses were conducted in R. The R script for extracting the relevant data is publicly available.[18]

## Results

**Fig 1:**
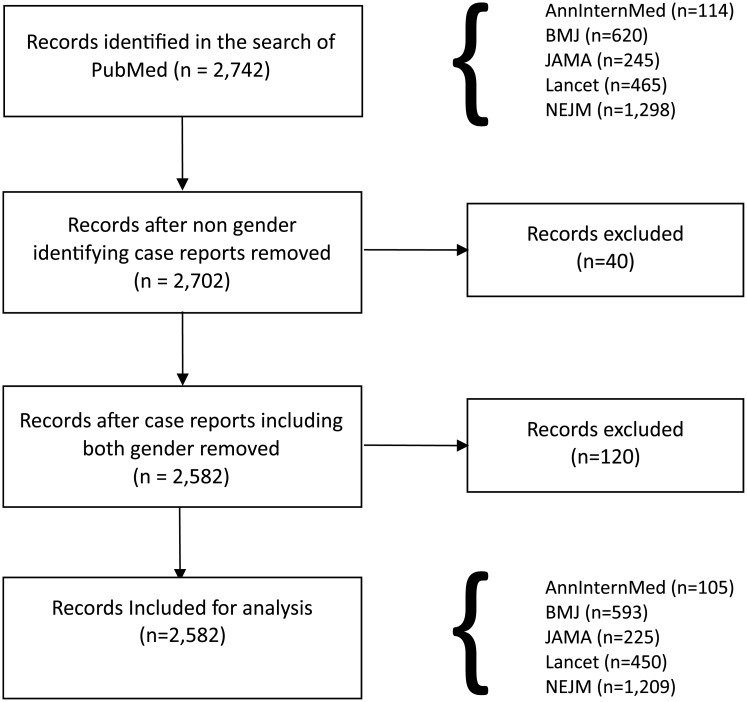
Study flow chart of clinical case report selection.

The initial search identified 2,742 case reports. Forty (40) case reports were excluded because the gender of the patient was not identified; and a further 120 case reports were excluded because they related to patients of both sexes. The final sample of case reports was 2,582, accounting for 94.2% of the initially identified reports. The flow diagram of case report inclusion is shown in Fig 1.

Of the 2,582 case reports, just under half (46.8%) appeared in the *New England Journal of Medicine (NEJM)*, 23% appeared in the *British Medical Journal (BMJ)*, 17.4% appeared in *The Lancet (Lancet)*, 8.7% appeared in the *Journal of the American Medical Association (JAMA)*, and 4.1% appeared in the *Annals of Internal Medicine (AnnInternMed)*. Of the case reports, 1,168 (45.2%) involved female patients and 1,414 (54.8%) involved male patients. Table 1 shows the sex distribution of the case reports for each journal.

**Table 1:** A word frequency analysis of the MeSH text associated with the 2,582 case reports aggregated into four categories for each gender: body/organ, disease, symptom, and investigation/treatment.

	Body / Organ				Disease			
**Rank**	**Female**	**Freq**	**Male**	**Freq**	**Female**	**Freq**	**Male**	**Freq**
1	Blood	155	Blood	161	Neoplasms	185	Neoplasms	195
2	Skin	68	Skin	110	Infections	86	Infections	131
3	Lung	68	Lung	105	Pregnancy	64	Injuries	83
4	Abdomen	62	Abdomen	78	Pulmonary	56	Pulmonary	49
5	Brain	60	Brain	71	Diabetes	34	Renal failure	40
6	Heart	49	Kidney	63	Injuries	32	Diabetes	38
7	Artery	46	Bone	61	Hypertension	29	HIV	37
8	Bone	45	Heart	58	Fractures	24	Hypertension	37
9	Kidney	41	Liver	53	Thrombosis	23	Infarction	36
10	Breast	38	Artery	49	Renal Failure	21	Fractures	34
	Symptom				Investigation/Treatment			
**Rank**	**Female**	**Freq**	**Male**	**Freq**	**Female**	**Freq**	**Male**	**Freq**
1	Pain	119	Pain	133	Radiography	262	Tomography	186
2	Dyspnea	63	Fever	87	Tomography	119	X-Ray	176
3	Fever	42	Exanthema	47	X-Ray	112	Surgery	143
4	Headache	34	Dyspnea	44	Surgery	101	MRI	113
5	Inflammation	31	Hemorrhage	38	MRI	95	Biopsy	65
6	Hemorrhage	29	Edema	34	Ultrasonography	95	Electrocardiography	48
7	Anemia	26	Headache	29	Biopsy	48	Ultrasonography	42
8	Edema	25	Anemia	27	Transplanation	31	Transplantation	37
9	Exanthema	21	Inflammation	27	Echocardiography	30	Angiography	35
10	Vomiting	19	Fatigue	20	Angiography	28	Echocardiography	35

A series of logistic regression models were developed with the sex in the case report as a dichotomous outcome variable, and the journal as a potential explanatory factor for observed variation in the proportion of female case reports. A random intercepts logistic regression model was developed to estimate the variance associated with journal as a random intercept. The associated variance was extremely small (4.0x10^-14^) and the inclusion of the journals as a fixed effect in a second model reduced the variance to zero. A Wald test for inclusion of the fixed effects showed no significant difference between the two models (Chi-sqr = 4.178, df = 4, p = 0.382) indicating that the data from the 2,582 case reports could be directly pooled.

**Fig 2:**
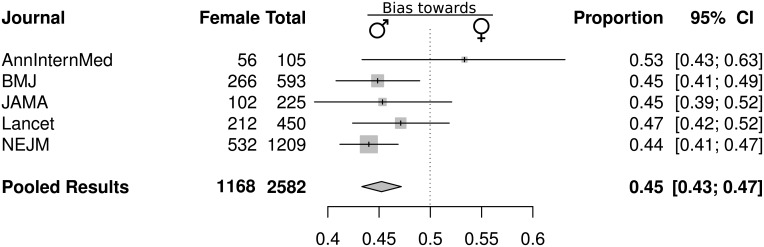
The gender bias in clinical case reports among the “big five” journals.

Fig 2 shows a combined plot and table of the data associated with each journal; the number of female case reports and the total; and the 95% confidence interval surrounding the individual journal estimate. The pooled result is shown last.

Squares and the horizontal lines represent the measures of effect, e.g. the proportion of case reports about women, and associated confidence intervals for each journal, and the diamond indicates the summary measure.

The *NEJM* (.44; 95%CI:.41–.47) and BMJ (.45; 95%CI:.41–.49) were the only two journals that showed a statistically significant bias against case reports of female patients. The *Annals of Internal Medicine* was the only journal that had a point estimate in the direction of a bias against male patients (.53; 95%CI:.43–.63). The *Annals of Internal Medicine*, however, published relatively few case reports (n = 105) and the confidence intervals were consequently wide. The remaining two journals had point estimates in the direction of a bias against female patients; however, neither of the individual estimates were significantly different from 0.5. In the pooled analysis, there was a statistically significant gender bias in case reports against female patients (0.45; 95%CI: 0.43–0.47). The 95% Confidence intervals of the individual journals’ estimates including the *Annals of Internal Medicine* overlapped the pooled estimate indicative of a gender bias.

To clarify that the gender bias was not a “*NEJM* problem” we recalculated the pooled estimate with only four of the journals, excluding the *NEJM* data (n = 1,444). The bias remains, albeit slightly smaller, and with wider confidence intervals because of the smaller sample size (.46; 95%CI:.437–.497, p = .006).

### Text analysis

Table 1 shows the top 10 most frequently used words related to body/organ, disease, symptom, or investigation/treatment in the MeSH of the 2,582 case reports. The frequencies were calculated separately for case reports of male and female patients.

There is some variation by sex in the rank order of words / conditions used in each category; nonetheless, there is remarkable consistency. In the body/organ category for example, the top five words for males and females fall in the same rank order. In the top 10 body/organ words, the only difference by gender is the inclusion of “breast” for women (Rank 10) and “liver” for men (Rank 9). There is similarly strong consistency in the disease category (“neoplasm” and “infection” ranked first and second for both groups). Investigation/treatment words relate largely to imaging with the exceptions being “biopsy”, “transplantation” and “surgery” for both males and females.

## Discussion

There were 10% more case reports about male patients than female patients identified in the review. What appears to be a relatively small effect in cross-section should really be seen as a potentially accumulating effect over time; and small accumulating effects can have a substantial impact.[19]

The concern is that a bias in clinical perception and decision making may be reinforced with continuous exposure towards an over representation of case reports involving male patients—supporting historical biases in clinical medicine and clinical research.[20] Medicine in males may be regarded as more main stream, more interesting, more indicative of what is a normal disease process. Because the case reports have become an integral part of medical education, the risks of gender biased exposure to “interesting clinical medicine” is potentially compounded, particularly in junior clinicians who are still laying down the matrix of expertise.[21] Unfortunately, the nature of these kinds of influences on decision making are likely to be subtle, hard to recognise in one's own decision making, and even when one is alert to the bias, formidable to overcome.[3]

What is surprising, is that the textual analysis of the MeSH words identified a remarkable similarity between case reports about male and female patients. If similar kinds of clinical cases are catching the eyes of authors and editors, is there a need to represent those clinical cases with a gender imbalance? Are there inherently more interesting characteristics in male than in female patients?

It is impossible to know the source of the observed bias, but there are three potential explanations. First, the apparent bias is an artifact of random noise in the data. This seems unlikely given the narrowness of the confidence interval around the estimate, but it is certainly a possibility. The second possibility is that there is an authorial bias. Authors of case reports are submitting more case reports about male patients than female patients to journals, and the journals are publishing them in the gender ratio with which they are received. The third possibility is that there is an editorial bias. Authors submit a gender balance of equally meritorious case reports and editors are more likely to approve for publication those reports about male patients. The extension of explanation two and three is that some interplay arises between an authorial and an editorial bias.

The sex and gender equity in research guidelines (SAGER) developed by the European Association of Science Editors were designed primarily to guide authors in preparing their manuscripts, but they are also useful for editors, as gatekeepers of science, to integrate assessment of sex and gender into all manuscripts as an integral part of the editorial process.”[2] In developing guidelines to ensure that gender is appropriately reflected in research the focus is on the individual article.[2] With respect to case reports, the role of the editor needs to extend beyond the impossible task of identifying bias in any specific case report, and should include an overview of the journal's corpus of published work.

### Strengths and limitations

This is the first study of its kind reported in the medical literature, and with the availability of the data extraction script other researchers can readily update and adapt the approach.[18] In conducting this study we relied on automated tools for database interrogation and text mining. It may be that when each case report is understood in its complete context an apparent bias is completely explicable and excusable, or disappears. This needs to be balanced against the capacity of automated techniques to flag issues of potential concern and interest.

There are many internal and general medicine journals, and the five journals we examined may not reflect the gender balance of case reports across the entire population of journals. The results do, however, reflect the imbalance in the the most prestigious of the journals (IF>16), and therefore in the journals with the greatest potential to influence the thinking of clinicians and researchers. This also raises questions about the potential gender imbalance in specialist journals. It is to be expected that some specialties will have a gender imbalance (e.g., obstetrics), but what of cases reports in journals of cardiology, gastroenterology, or neurology?

### Conclusion

This is the first study to review gender bias in clinical case reports, and the results raise questions about the existence of other group biases (e.g., ethnicity or race bias), whether some clinical areas are more prone towards a bias, and whether the source of the bias lies with the authors or the editors. The study also provides a further benchmark in the assessment of progress towards gender parity in science and medicine. It is important that journals are aware of the inherent bias and the implicit message this may convey to the medical community. Journal Editors are well placed to monitor the bias over time and make affirmative decisions to reduce it, and a reduction in the gender bias can only improve the overall quality and value of the case reports to the readership.
